# Solitary vertebral metastatic glioblastoma in the absence of primary brain tumor relapse: a case report and literature review

**DOI:** 10.1186/s12880-020-00488-x

**Published:** 2020-07-31

**Authors:** Zu-Gui Li, Min-Ying Zheng, Qi Zhao, Kai Liu, Jia-Xing Du, Shi-Wu Zhang

**Affiliations:** 1 Graduate School, Tianjin University of Traditional Chinese Medicine, Tianjin, 301617 China; 2 Department of Pathology, Tianjin Union Medical Center, Tianjin, 300121 China; 3 Nankai University School of Medicine, Nankai University, Tianjin, 300070 China

**Keywords:** Case report, Extracranial metastasis, Solitary vertebral metastasis, Glioblastoma, Spinal neoplasm

## Abstract

**Background:**

Metastatic glioblastoma presenting as a solitary osteolytic cervical vertebral mass without primary brain tumor relapse is extremely rare with only 1 reported case in the literature. Because of its rarity, it can be easily overlooked and misdiagnosed, posing a diagnostic dilemma.

**Case presentation:**

A 51-year-old man with right temporal glioblastoma was initially treated by tumor resection, radiotherapy and chemotherapy. Eighteen months after surgery, he was readmitted with complaints of neck pain for 2 weeks. Follow-up magnetic resonance imaging (MRI) and fluorodeoxyglucose (FDG) positron emission tomography/computed tomography (PET/CT) revealed a solitary FDG-avid osteolytic lesion in the 4th cervical vertebral body without other abnormal FDG-uptake in the body and in the absence of local recurrence at the resection cavity. Because of the sudden worsening situation and intractable neck pain, the patient underwent tumor resection. Postoperatively, the pain was obviously reduced and the situation was improved. Interestingly, the immunohistochemical findings of glial fibrillary acidic protein (GFAP) indicated the characteristic of metastatic glioblastoma, despite that the histopathological findings of Hematoxylin & Eosin (H&E) staining was suspicious of osteoclastoma. According to the clinical history, imaging findings, pathological and immunohistochemical results, a final diagnosis of solitary vertebral metastasis from glioblastoma without central nervous system (CNS) relapse was confirmed. Then, the patient received radiotherapy on spine and adjuvant chemotherapy with temozolomide. However, he died suddenly 2 months after the tumor resection, nearly 21 months after the initial diagnosis.

**Conclusion:**

We emphasize that metastatic glioblastoma should be considered in the differential diagnosis of a solitary FDG-avid osteolytic vertebral mass on PET/CT. And the diagnosis of extracranial metastasis (ECM) from glioblastoma can be achieved through clinical history, imaging findings, pathological examination, and immunohistochemical staining with GFAP.

## Background

Glioblastoma is the most aggressive primary brain tumor in adults that accounts for nearly 20% of all primary malignant CNS tumors. Despite the standard treatment with surgery, radiotherapy, and chemotherapy with temozolomide, the median survival time for glioblastoma is only 15 months [1]. While glioblastoma is notable for local recurrence and invasion, ECM is exceedingly rare, occurring in less than 2% of patients [2], as compared with a frequency of 10% of CNS metastasis from other tumors [3].

ECM frequently occurred in regional lymph nodes (51%), lungs and pleura (60%), bones (31%), liver (22%), and other metastatic sites including the soft tissues, the spleen, the kidney, the orbit and the heart [4–7]. Regarding bone metastases of glioblastoma, they usually present as a multiple extracranial spread associated with metastatic involvement of other organ systems [6, 8]. ECM of glioblastoma presenting as a solitary bone metastasis is extremely rare. To best of our knowledge, solitary extracranial vertebral metastasis in the absence of CNS relapse and without other metastatic sites in the body is exceedingly rare with only 1 reported case in the literature [9].

Because of its rarity, it can be easily overlooked and misdiagnosed as various conditions, posing a diagnostic dilemma. Herein, we present an exceedingly rare case of solitary vertebral metastatic glioblastoma in the absence of primary brain tumor relapse to highlight that unexpected ECM should not be ignored in caring for the patients with a history of glioblastoma even though the primary brain tumor is well-controlled.

## Case presentation

**Fig. 1:**
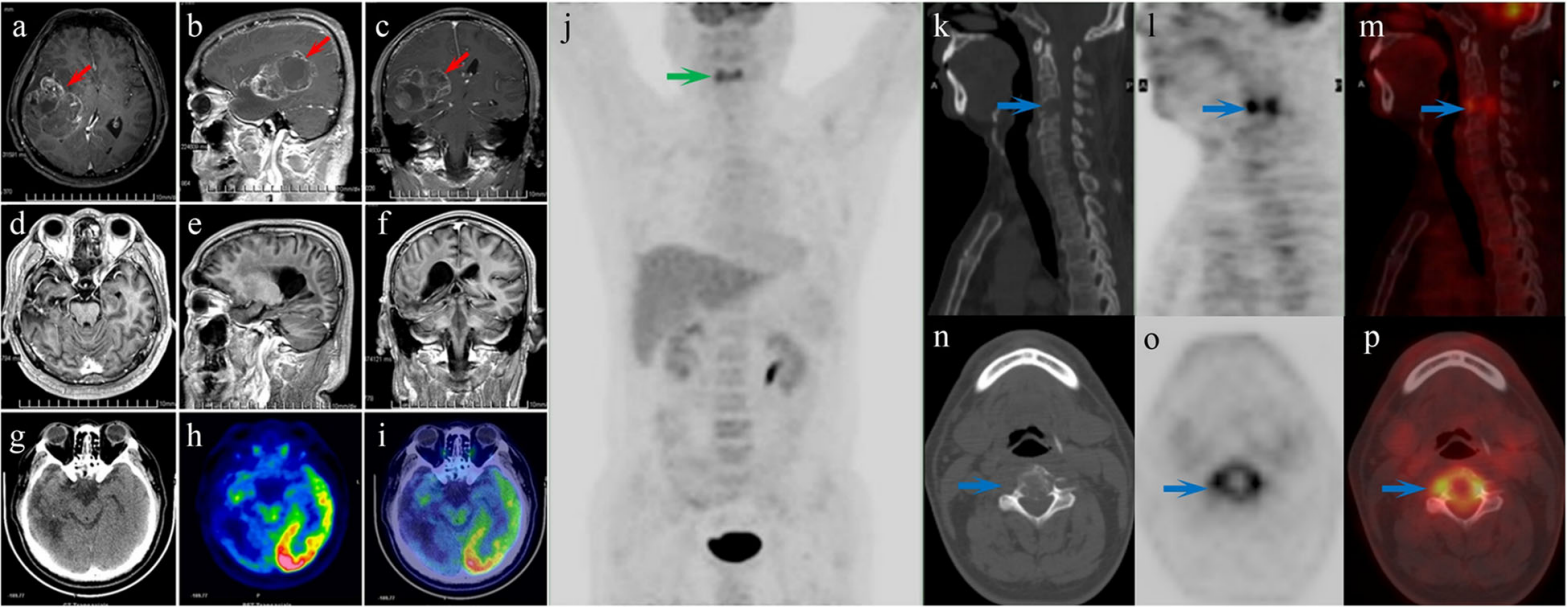
**a**-**c** Presurgical T1-weighted MR images showed a large, lobulated, and ill-defined 7.8 cm × 5.3 cm × 4.0 cm mass with inhomogeneous enhancement in the right temporal lobe (red arrows). The involvement of right lateral ventricular was also observed. **d**-**f** Follow-up postcontrast MR and (**g**-**i**) brain FDG PET /CT images indicated the postoperative changes without any signs of local recurrence at the resection cavity. (**j**) Whole-body FDG PET/CT anteroposterior 3-dimensional maximum intensity projection (3D-MIP) image showed a focal abnormal FDG-avid lesion in the cervical region without any other positive findings in the body (green arrow). (**k**-**p**) The selected sagittal and transaxial views of PET/CT images demonstrated a solitary intense FDG-avid osteolytic lesion associate with a compression fracture in the 4th cervical vertebral body (blue arrows). (**o**-**p**) Transaxial view of PET/CT images showed (blue arrow) increased FDG-uptake in the periphery of the mass with a photopenic center, the so-called “doughnut” sign

A 51-year-old man initially presented with a 3-week history of progressively worsening headache and left limb weakness, and with a 2-day history of nausea and vomiting. T1-weighted MR images showed a large, lobulated, and ill-defined 7.8 cm × 5.3 cm × 4.0 cm mass with inhomogeneous ring-like enhancement in the right temporal lobe (Fig. 1 a-c). A craniotomy was performed with tumor resection and the final pathological diagnosis was glioblastoma (WHO IV). Then, he received several cycles of radiotherapy and chemotherapy with temozolomide. Eighteen months after the surgical operation, he was readmitted with complaints of pain on neck for nearly 2 weeks. Follow-up contrasted brain MR imaging and whole-body FDG PET/CT imaging with a separate acquisition of the brain was performed to further evaluate the situation. The following contrasted brain MR imaging and brain FDG PET/CT indicated no signs of tumor recurrence at the resection cavity (Fig. 1 d-i). However, whole body PET/CT imaging demonstrated a solitary hypermetabolic osteolytic lesion with maximal standard uptake value (SUV_max_) of 8.0 in the 4th cervical vertebra associated with compression fracture (Fig. 1 j-p). FDG PET/CT images also demonstrated intense FDG-uptake in the periphery of the mass with a photopenic center, the so-called “doughnut” sign. (Fig. 1 o-p).

Despite the patient has a history of glioblastoma, it is a great challenge in making a diagnosis of solitary metastatic glioblastoma because of the rarity and the non-specific FDG PET/CT imaging findings. To narrow the differential diagnosis, laboratory tests including ESR, M-type protein, Bence-Jones protein were performed to help exclude the possibility of plasmacytoma. However, the results were normal. Meanwhile, cervical spine MRI and biopsy were suggested to further evaluate the cervical vertebral lesion. Unfortunately, the patient refused to have further examination because of the sudden worsening situation, especially the intractable neck pain. To alleviate the pain and to prevent progressive compression symptoms, the patient underwent tumor resection. Postoperatively, the pain and compression symptoms were obviously relived and the situation was improved.

**Fig. 2:**
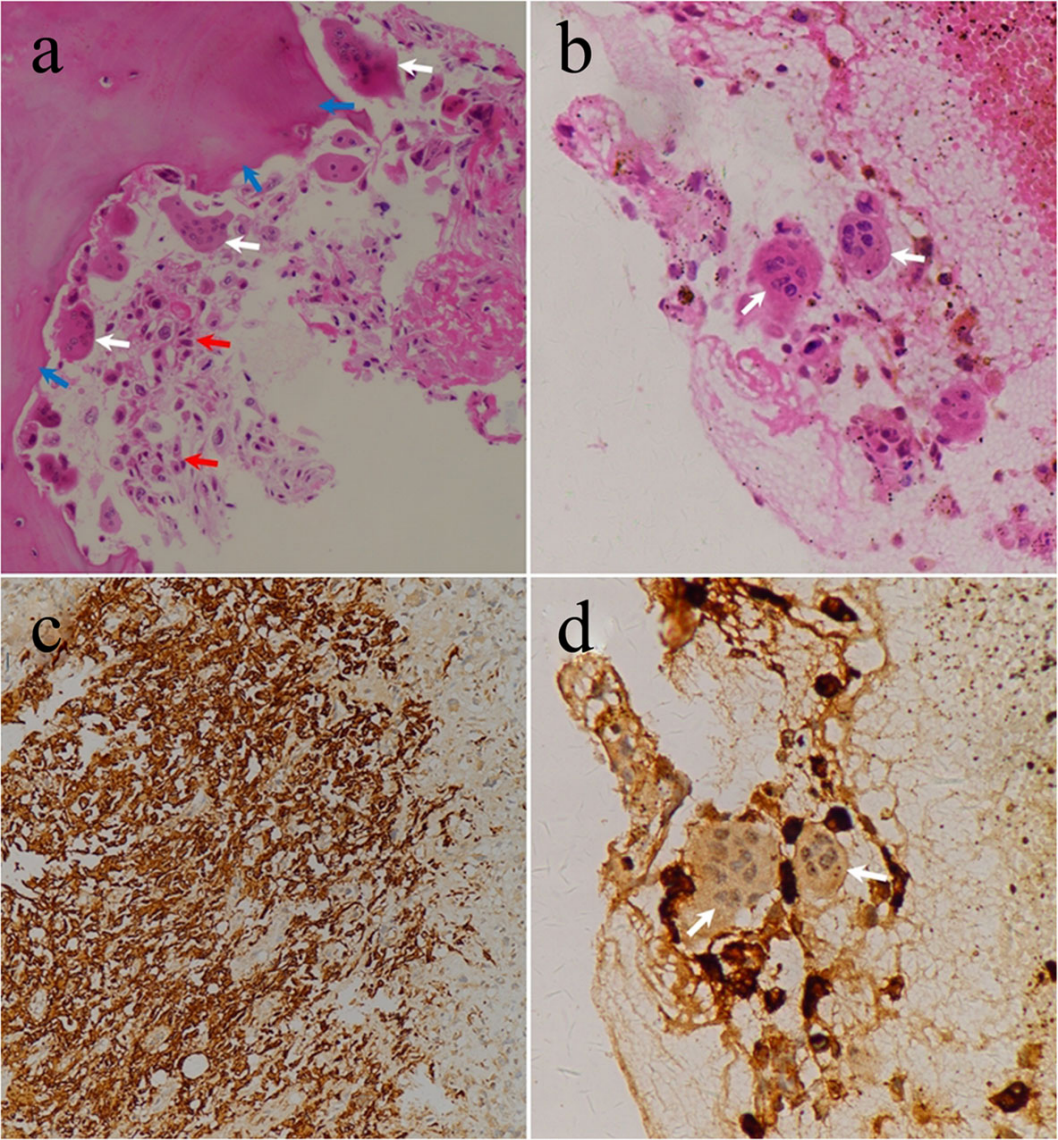
**a-b** Hematoxylin & Eosin staining of the tumor demonstrated atypical tumor cells with marked hyperchromatism, pleomorphism (red arrows). The presence of residual bony tissues (blue arrows) and the distinguishing characteristics of multinucleated giant cells in the tumor (white arrows) were also observed. **c**, **d** Immunohistochemical stains showed tumor cells were positive for GFAP, while the multinucleated giant cells were negative for GFAP (white arrows). These findings were consistent with the diagnosis of metastatic glioblastoma. ((**a**), (**b**), (**d**) magnification× 200; (**c**) magnification × 100)

The following histopathological findings of H&E staining indicated the presence of characteristic multinucleated giant cells in the tumor (Fig. 2 a, b), which was suspicious of osteoclastoma. Given the history of glioblastoma, immunohistochemical staining with GFAP was performed to exclude the possibility of metastatic glioblastoma. Interestingly, the immunohistochemical findings of GFAP were positive in bone specimens, which substantiated the diagnosis of ECM from glioblastoma (Fig. 2 c, d). Based on these results, a final diagnosis of solitary vertebral metastasis from glioblastoma without CNS relapse was confirmed. Then, he received radiotherapy on spine and adjuvant chemotherapy with temozolomide. Two months after cervical surgery, he was readmitted with complaints of 4-day history of progressive pain on neck. Then, he died suddenly due to respiratory insufficiency in only 2 weeks, nearly 21 months after the initial diagnosis.

## Discussion and conclusions

Albeit rare, ECMs from glioblastoma do occur, carrying a poor prognosis. The old notion that primary cerebral gliomas never metastasize outside the CNS has been challenged by the accumulating incidence of ECMs from various intracranial tumors. The reported frequency of ECM was only 0.44% in 1966, but the present incidence rate has raised to nearly 2% [4, 10]. It has been previously reported that the average time from initial diagnosis to spinal metastasis is 26.4 months, and the time from spine metastasis to death is only 10 months for patients diagnosed with glioblastoma [6]. In our case, the time from diagnosis to detection of ECM is approximately 18 months, but the time from ECM to death is only 2 months. Therefore, ECM may act as a crucial indicator of poor prognosis, and earlier diagnosis and treatment may improve the prognosis.

In clinical experience, it is a great challenge in the differential diagnosis of solitary cervical vertebral mass because of the overlapping imaging and clinical appearances. Clinical symptoms frequently consist of pain and neurologic symptoms due to compression of the spinal cord following vertebral compression fracture, which can be occurred in a wide variety of benign and malignant lesions on the spine. Moreover, solitary osteolytic cervical vertebral lesion with increased FDG-uptake is non-specific, it can be observed in various pathological conditions including metastasis, solitary plasmacytoma, osteoclastoma, lymphoma, aneurysmal bone cyst, eosinophilic granuloma, tuberculosis, and so on [11–22].

Given the history of glioblastoma, metastatic glioblastoma should be firstly included in the differential diagnosis. However, extracranial bone metastases from glioblastoma frequently present as a multiple extracranial spread with the involvement of other sites in the body [6, 8]. Solitary cervical vertebral metastasis is extremely rare with only 1 reported case in the literature [9]. Thus, it is not easy to make a diagnosis of solitary metastatic glioblastoma on FDG PET/CT because of its scarcity and the non-specific imaging features.

Following solitary spine metastasis, plasmacytoma is the most common malignancy of the spine in a mid aged or elderly patient. Radiologically, a solitary plasmacytoma usually presents as an osteolytic lesion without new bone formation, or as a sclerotic reaction [18, 19]. It frequently occurs in the thoracic vertebrae and lumbar spine, while cervical spine involvement is relatively rare [18]. In our case, although the negative results of laboratory tests including ESR, M-type protein, Bence-Jones protein may be helpful to exclude the possibility of plasmacytoma, but histologic confirmation of the diagnosis is necessary.

In our case, FDG PET/CT imaging also showed the “doughnut sign” with missing central but increased peripheral FDG uptake, indicating the diagnosis of osteoclastoma. However, the sign is also non-specific, it can be widely observed in other entities including bone cysts, aneurysmal bone cyst, capillary hemangioma, and other malignancies such as chondrosarcomas and teleangiectaic sarcomas, which are however uncommon in cervical spine [23–25]. Furthermore, the most common site of osteoclastoma occurrence is the distal femur, followed by the proximal tibia, distal radius, sacrum, and proximal humerus, while the spine is rarely involved [19, 20].

Primary bone lymphoma is very rare, comprising for less than 5% of extranodal lymphomas, and less than 1% of all non-Hodgkin lymphomas. Furthermore, primary bone lymphoma of spine is exceedingly rare, accounting for only 1.7% of all primary bone lymphoma. Primary bone lymphoma can be manifested with a FDG-avid solitary osteolytic lesion, but it is not unique [15, 21]. Thus, it is not easy to make the diagnosis of bone lymphoma on the basis of the non-specific FDG PET/CT imaging findings.

The aneurysmal bone cyst and eosinophilic granuloma can present with solitary localized osteolytic lesion with abnormal FDG uptake in spine [13]. However, they typically affect young patients [16, 22]. In adults with a solitary osteolytic lesion in the vertebral body, tuberculosis should be also considered despite of the rarity. Despite the CT imaging findings of a large osteolytic lesion surrounded by obvious bone sclerosis may favor a diagnosis of tuberculosis, it can be easily misdiagnosed with other diseases [17].

With some entities, the differential diagnosis of solitary bone lesion can be difficult even for an experienced bone tumor pathologist. In our case, H&E staining findings showed multinucleated giant-cells or osteoclast-like cells among tumor cells and it can be easily misdiagnosed as osteoclastoma without the imaging findings and clinical history. Considering the history of glioblastoma, immunohistochemical staining with GFAP, a specific marker for astrocytes, was performed to differentiate osteoclastoma from glioblastoma, since the latter is positive for GFAP. Interestingly, the following immunohistochemical results indicated that the tumor cells were positive for GFAP. Taken together, differential diagnosis of a solitary osteolytic metastatic glioblastoma in the spine can be a perplexing task. However, a definite diagnosis may be achieved through clinical history, imaging findings, pathological examination, and immunohistochemical staining with GFAP.

To date, possible mechanisms triggering ECMs from glioblastoma remain elusive. Some reports ascribed the rarity of ECM to the short survival of the disease, and others held that the rarity of ECM is due to the lack of lymphatics, relatively impassable dura, extracellar matrix, and tough basement membrane surrounding brain blood vessels [26]. Generally, ECM of primary brain tumors is considered to spread in any of three routes: seeding through the cerebral fluid pathway, local invasion, or spreading remotely through lymphatic and blood vessels [27]. Regarding cervical vertebral metastasis in our case, the tumor cells may enter the Batson plexus and propagated in the cerebrospinal fluid. Moreover, some connection may exist between the meningeal and craniocervical venous system, which can join the internal vertebral venous plexus. The internal vertebral venous plexus flows back to the anterior and posterior surface of the cervical vertebrae, which may give rise to a solitary cervical vertebral metastasis from glioblastoma [6, 9]. Risk factors for ECM were widely described which included a previous craniotomy, stereotactic biopsies, ventricular systemic shunting, high-grade tumor history, young age, radiation therapy, prolonged survival time, tumor recurrence and even a sarcomatous component [28, 29]. It is also reported that radiotherapy may induce sarcomatous metaplasia of glial cells and help glioblastoma acquire the necessary extracellar matrix proteins for vascular invasion and systemic metastases to distant extracranial sites [30]. Thus, in our case, craniotomy, radiotherapy and chemotherapy could be considered influencing factors in the formation of ECMs.

Moreover, the mechanisms responsible for osteolytic metastasis of glioblastoma may involve bidirectional interactions between brain tumor cells and bone. According to the century-old ‘seed and soil’ hypothesis initially established by Paget [31], glioblastomas may harbor such characteristics that enable them to grow in bone, and the bone microenvironment may provide a fertile soil on which to grow. In this case, the presence of multinucleated giant cells with negative GFAP expression may suggest the coexistence of bone-derived osteoclast and the glioblastoma cells, which may sustain the “seed and soil” hypothesis in explaining interactions between brain tumor cells and bone.

Despite the above-mentioned possible mechanisms of ECM, the underlying genetic and molecular mechanism should be further investigated. It was previously suggested that the metastatic potential of GBM might be related to TP53 gene mutations and the emergence of neoplastic subclones [32]. Moreover, overexpression of insulin-like growth factor binding protein-1(IGFBP2) and functional deficiency of DNA-dependent protein kinase proteins may promote tumor progression in glioblastoma [33]. It was also indicated that increased levels of the matrix metalloproteinase (MMP), active gelatinase-A have been revealed in glioblastoma with ECMs, as compared with glioblastomas without ECMs [34]. Regarding ECM of other primary CNS tumors, detection of molecular alternations such as combined deletion of the 1p and 19p chromosomal arms, hypermethylation of methyl guanine methyl transferase (MGMT) promoter, and phosphatase and tensin homolog deleted on chromosome ten (PTEN) exon mutations my help elucidate which subtypes of anaplastic oligodendroglioma have a trendency to develop ECMs [35].

So far, no standard treatment for ECM exists. A report indicates that aggressive therapy is not suggested in metastatic glioblastoma because of poor prognosis [36], but another report suggested that systemic treatment of ECM can prolong survival time and improve clinical conditions [37]. Although previous reports indicated that patients with solitary bone metastasis have a more favorable survival outlook than patients with multiple bone metastases [38, 39], our case report suggested that tumor resection followed by radiotherapy, and chemotherapy for solitary vertebral metastasis appears to have little impact on prolonging the patient’s survival. Thus, the surgical treatment mainly aims to preserve function, reduce patient’s pain and to improve quality of life [40]. The optimal treatment for ECM should be further investigated with a wide inclusion of patients.

Taken together, our case is unique in that rare conditions such as extracranial metastasis from glioblastoma in absence of intracranial tumor relapse, presenting as a solitary osteolytic metastasis in cervical vertebra, and the presence of multinucleated giant cells in histological H&E examination can be occurred in the same patient, simultaneously. Herein, we present this rare case of solitary vertebral metastatic glioblastoma to emphasize that metastatic glioblastoma should be considered in making a differential diagnosis of a solitary FDG-avid osteolytic vertebral mass on PET/CT. And we also emphasize that the diagnosis of ECM from glioblastoma can be achieved through the detail clinical history, imaging findings, pathological examination, and immunohistochemical staining with GFAP.

## Data Availability

The datasets used and analysed during the current study are available from the corresponding author on reasonable request.

## Abbreviations

MRI: Magnetic resonance imaging
FDG: Fluorodeoxyglucose
PET/CT: Positron emission tomography/computed tomography
CT: Computed tomography
GFAP: Glial fibrillary acidic protein
H&E: Hematoxylin & eosin
CNS: Central nervous system
ECM: Extracranial metastasis
SUV_max_: Maximal standard uptake value
IGFBP2: Insulin-like growth factor binding protein-1
MMP: Matrix metalloproteinase
MGMT: Methyl guanine methyl transferase
PTEN: Phosphatase and tensin homolog deleted on chromosome ten
3D-MIP: 3-dimensional maximum intensity projection
